# Reliability assessment of ultrasound muscle echogenicity in patients with rheumatic diseases: Results of a multicenter international web-based study

**DOI:** 10.3389/fmed.2022.1090468

**Published:** 2023-01-17

**Authors:** Andrea Di Matteo, Erica Moscioni, Maria Giovanna Lommano, Edoardo Cipolletta, Gianluca Smerilli, Sonia Farah, Carla Airoldi, Sibel Zehra Aydin, Andrea Becciolini, Karina Bonfiglioli, Marina Carotti, Greta Carrara, Tomas Cazenave, Davide Corradini, Micaela Ana Cosatti, Juan Josè de Agustin, Giulia Maria Destro Castaniti, Marco Di Carlo, Eleonora Di Donato, Luca Di Geso, Ashley Elliott, Daniela Fodor, Francesca Francioso, Alessandra Gabba, Cristina Hernández-Díaz, Rudolf Horvath, Jana Hurnakova, Diogo Jesus, Josefina Marin, Maria Victoria Martire, Riccardo Mashadi Mirza, Marco Massarotti, Alice Andreea Musca, Jagdish Nair, Tadashi Okano, Ioannis Papalopoulos, Javier Rosa, Marcos Rosemffet, João Rovisco, Davide Rozza, Fausto Salaffi, Crescenzio Scioscia, Carlo Alberto Scirè, Maria-Magdalena Tamas, Shun Tanimura, Lucio Ventura-Rios, Catalina Villota-Eraso, Orlando Villota, Paraskevi V. Voulgari, Florentin Ananu Vreju, Gentiana Vukatana, Johana Zacariaz Hereter, Anna Zanetti, Walter Grassi, Emilio Filippucci

**Affiliations:** ^1^Rheumatology Unit, Department of Clinical and Molecular Sciences, “Carlo Urbani” Hospital, Polytechnic University of Marche, Ancona, Italy; ^2^Leeds Institute of Rheumatic and Musculoskeletal Medicine, University of Leeds, Leeds, United Kingdom; ^3^Hospital Provincial, Rheumatology, Rosario, Argentina; ^4^Ottawa Hospital Research Institute, University of Ottawa, Ottawa, ON, Canada; ^5^Internal Medicine and Rheumatology Unit, Department of Medicine, Azienda Ospedaliero-Universitaria di Parma, Parma, Italy; ^6^Hospital das Clínicas da Faculdade de Medicina da Universidade de São Paulo, São Paulo, SP, Brazil; ^7^Department of Radiology, Ospedali Riuniti, Università Politecnica delle Marche, Ancona, Italy; ^8^Epidemiology Unit, Italian Society of Rheumatology, Milan, Italy; ^9^Rheumatology Unit, Instituto de Rehabilitación Psicofísica, Buenos Aires, Argentina; ^10^Rheumatology Unit, University Clinic AOU Cagliari, Monserrato, CA, Italy; ^11^CEMIC, Centro de Educación Médica e Investigaciones Médicas “Norberto Quirno”, Buenos Aires, Argentina; ^12^Rheumatology Unit, Vall d’Hebron Hospital Universitari, Vall d’Hebron Barcelona Hospital Campus, Barcelona, Spain; ^13^Department of Health Promotion, Mother and Child Care, Internal Medicine and Medical Specialties, Rheumatology Section, University of Palermo, Palermo, Italy; ^14^Department of Internal Medicine, Ospedale Madonna del Soccorso, San Benedetto del Tronto, Marche, Italy; ^15^Centre for Experimental Medicine, Queen’s University Belfast, Belfast, United Kingdom; ^16^2nd Department of Internal Medicine, “Iuliu Hatieganu” University of Medicine and Pharmacy, Cluj-Napoca, Romania; ^17^Local Health Unit (ASL), Samugheo, OR, Italy; ^18^Local Health Unit (ASL), Orosei, NU, Italy; ^19^División de Reumatología, Instituto Nacional de Rehabilitación “Luis Guillermo Ibarra Ibarra”, Mexico City, Mexico; ^20^Department of Paediatric and Adult Rheumatology, University Hospital Motol, Prague, Czechia; ^21^Department of Rheumatology, Centro Hospitalar de Leiria, Leiria, Portugal; ^22^Hospital Italiano de Buenos Aires, Buenos Aires, Argentina; ^23^San Roque Hospital, La Plata, Argentina; ^24^Department of Radiology, A. O. Ospedali Riuniti Marche Nord, Pesaro, Italy; ^25^Department of Rheumatology, University Hospitals Dorset NHS Foundation Trust, Christchurch Hospital, Christchurch, United Kingdom; ^26^Department of Rheumatology, Colentina Clinical Hospital, Bucharest, Romania; ^27^Department of Rheumatology, Liverpool University Hospitals Foundation Trust, Liverpool, United Kingdom; ^28^Department of Orthopedic Surgery, Osaka Metropolitan University Graduate School of Medicine, Osaka, Japan; ^29^Department of Rheumatology, Clinical Immunology and Allergy, University Hospital of Heraklion, Heraklion, Greece; ^30^Department of Rheumatology, Centro Hospitalar e Universitário de Coimbra, Coimbra, Portugal; ^31^Rheumatology Unit, Department of Emergency and Organ Transplants (DETO), University of Bari, Bari, Italy; ^32^Department of Rheumatology, “Iuliu Hatieganu” University of Medicine and Pharmacy, Cluj-Napoca, Romania; ^33^Department of Rheumatology, Hokkaido Medical Center for Rheumatic Diseases, Sapporo, Japan; ^34^IPS Servicio Integral de Reumatología e Inmunología Doctor Orlando Villota, Pasto, Colombia; ^35^Division of Rheumatology, Fundación Hospital San Pedro, Pasto, Colombia; ^36^Department of Rheumatology, School of Health Science, Faculty of Medicine, University of Ioannina, Ioannina, Greece; ^37^Department of Rheumatology, University of Medicine and Pharmacy of Craiova, Craiova, Romania; ^38^Rheumatology Unit, IRCCS Policlinico S. Orsola-Malpighi, Bologna, Italy

**Keywords:** muscle echogenicity, musculoskeletal ultrasound, sarcopenia, reliability, rheumatic diseases

## Abstract

**Objectives:**

To investigate the inter/intra-reliability of ultrasound (US) muscle echogenicity in patients with rheumatic diseases.

**Methods:**

Forty-two rheumatologists and 2 radiologists from 13 countries were asked to assess US muscle echogenicity of quadriceps muscle in 80 static images and 20 clips from 64 patients with different rheumatic diseases and 8 healthy subjects. Two visual scales were evaluated, a visual semi-quantitative scale (0–3) and a continuous quantitative measurement (“VAS echogenicity,” 0–100). The same assessment was repeated to calculate intra-observer reliability. US muscle echogenicity was also calculated by an independent research assistant using a software for the analysis of scientific images (ImageJ). Inter and intra reliabilities were assessed by means of prevalence-adjusted bias-adjusted Kappa (PABAK), intraclass correlation coefficient (ICC) and correlations through Kendall’s Tau and Pearson’s Rho coefficients.

**Results:**

The semi-quantitative scale showed a moderate inter-reliability [PABAK = 0.58 (0.57–0.59)] and a substantial intra-reliability [PABAK = 0.71 (0.68–0.73)]. The lowest inter and intra-reliability results were obtained for the intermediate grades (i.e., grade 1 and 2) of the semi-quantitative scale. “VAS echogenicity” showed a high reliability both in the inter-observer [ICC = 0.80 (0.75–0.85)] and intra-observer [ICC = 0.88 (0.88–0.89)] evaluations. A substantial association was found between the participants assessment of the semi-quantitative scale and “VAS echogenicity” [ICC = 0.52 (0.50–0.54)]. The correlation between these two visual scales and ImageJ analysis was high (tau = 0.76 and rho = 0.89, respectively).

**Conclusion:**

The results of this large, multicenter study highlighted the overall good inter and intra-reliability of the US assessment of muscle echogenicity in patients with different rheumatic diseases.

## Introduction

Sarcopenia is a muscle disease that is characterized by low muscle mass (main criteria), reduced muscle strength and impaired physical performance (1, 2). Sarcopenia is regarded as the biological foundation of frailty. Both these conditions have been demonstrated to have an association with increased adverse health outcomes such as falls, hospital admission, and mortality (3). In a recent study on 400 patients with rheumatoid arthritis (RA), a significant association was found between sarcopenia and multiple RA-related comorbidities, including obesity, dyslipidemia, diabetes and chronic obstructive pulmonary disease (4).

While “primary” sarcopenia reflects age-related changes in muscle mass, strength and function, “secondary” sarcopenia may occur in relatively young patients with inflammatory diseases, such as RA, mainly as the consequence of chronic systemic inflammation, use of medications (e.g., steroids) and patients’ reduced mobility (5–7).

Magnetic resonance imaging (MRI), computed tomography (CT), bioelectrical impedance analysis and dual-energy x-rays absorptiometry (DXA) are regarded as the reference imaging tests for the assessment of sarcopenia (8). Several studies have also highlighted the very promising role of ultrasound (US) as a reference method for the evaluation of sarcopenia-related muscle involvement in elderly populations (9) and, to a lesser extent, in patients with rheumatic diseases (10). Muscle US based measurements have shown a strong correlation with MRI, CT and DXA based evaluations (11–13). In addition, US has been proven accurate for the evaluation of muscle quantity and quality in validation studies on cadavers (14, 15).

As acknowledged by the SARCopenia through UltraSound (SARCUS) working group (i.e., a Sarcopenia Special Interest Group of the European Geriatric Medicine Society), the use of US in sarcopenia is promising but limited mainly by lack of standardization and data supporting the reliability of this imaging tool (16).

The US measurement of muscle mass (i.e., muscle thickening) is regarded as the “traditional” US method for the diagnosis of sarcopenia (17). However, no clearly defined US cut-offs for the diagnosis of sarcopenia (neither for MRI nor CT) have been established (18). In addition, a reduction of muscle mass is only one of the aspects that characterize the process of sarcopenia-related muscle degradation, and arguably the one that is most influenced by aging (19).

Also “qualitative” changes of muscle architecture (i.e., increased muscle echogenicity due to fatty replacement or fibrosis of muscle tissue) have emerged as important US features of sarcopenia (20). Previous studies have demonstrated that an increased US muscle echogenicity, notwithstanding preserved muscle mass, is a relevant and accurate measure of sarcopenia-related muscle deterioration (i.e., reduced muscle quality) (21).

In last years, rheumatologists have been attracted by the promising role of US in the assessment of sarcopenia (or “sarcopenia spectrum”) in patients with rheumatic diseases (22–25). The “early” detection of sarcopenia in patients with rheumatic diseases may raise important implications for the management of these patients, including the adoption of regular exercise and/or the use of medications and supplements (26).

In a very recent study, our research group has proposed a new US protocol for the evaluation of various aspects of sarcopenia-related muscle involvement (“multimodal ultrasound”), including muscle mass, muscle echogenicity/quality and muscle stiffness using shear-wave elastography (27). In this study, a four-grade US visual semi-quantitative scale for the assessment of muscle echogenicity was developed. Unlike the measurement of muscle mass, this US semi-quantitative scale showed the ability to discriminate between systemic lupus erythematosus (SLE) patients and healthy subjects. In addition, an increased US muscle echogenicity was significantly associated with patients’ reduced muscle strength and low physical performance, thus emerging as a valuable tool for the early detection of muscle deterioration associated with sarcopenia in patients with SLE and, potentially, in patients with rheumatic diseases (27).

Beside the potential clinical implications of the recently proposed US semi-quantitative scale for muscle echogenicity, an equally important requisite for the application of this imaging method in clinical practice is the grade of consistency/agreement between different individuals (inter-reliability), and within the same individual on different occasions (intra-reliability), in the reading and interpretation of such scale.

Therefore, the main objective of the current study was to explore, in a large group of physicians (mostly rheumatologists) who routinely perform musculoskeletal (MSK) US, the inter and intra-reliability of the visual US semi-quantitative scale for muscle echogenicity which was recently developed by our research group. The inter and intra-reliability of a second quantitative visual scale for muscle echogenicity (from 0 to 100, VAS echogenicity) was also investigated, as well as the association between these two US visual scales and their correlation with an image-processing program that uses histogram analysis to calculate pixel gray scale intensity in a region of interest (ROI).

## Materials and methods

Rheumatologists and MSK radiologists who had a training period in MSK US at the “Rheumatology Clinic” of the “Carlo Urbani” Hospital, Jesi, Ancona, Italy, were invited to participate in this web-based exercise. A detailed description of this research group has been recently published (28).

B-mode images and clips (5–10 s long) of the quadriceps muscle (i.e., rectus femoris and vastus intermedius muscles) were collected by 2 rheumatologists with 10 (ADM) and 4 years (GS) of experience in MSK US. The images and clips were obtained using a transverse approach in 64 patients with different rheumatic conditions (16 systemic sclerosis, 15 axial spondyloarthritis, 10 RA, 9 SLE, 6 osteoarthritis, 4 fibromyalgia, 2 gout, 2 calcium pyrophosphate deposition disease), with no current symptoms suggesting inflammatory myositis (nor a previous diagnosis of inflammatory myositis/neuromuscular disease), who attended the out-patient clinic of Rheumatology Unit, Jesi (Ancona), and 8 healthy subjects (staff members of the “Carlo Urbani” hospital). In 24 out of 64 rheumatic patients and in 4 out of 8 healthy subjects, a bilateral acquisition of the quadriceps muscle was obtained (left and right quadriceps muscle). Therefore, the images and clips composing the final US dataset were acquired from 100 different quadriceps muscles. Creatine phosphokinases were not systematically obtained in the current study. However, patients with an increased creatine phosphokinase recorded at least once in the 6 months preceding the enrollment, and patients/healthy subjects who had intense physical activity in the preceding 4 weeks, were excluded from the study. The mean age, gender, and body mass index (BMI) were 60.3 ± 13.7 years, 64.1% female, and BMI: 25.7 ± 5.4 in rheumatic patients and 37.2 ± 6.5 years, 50.0% females, and BMI: 24.6 ± 4.0 in healthy subjects. Fifteen out of 64 (23.4%) rheumatic patients were on corticosteroids (≥5 mg of oral prednisolone equivalents).

The final dataset included 80 static images and 20 clips. Images and clips were exported from the US machine and saved in JPEG and AVI format, respectively. The two rheumatologists (ADM and GS) developed the final images and clips dataset balancing the prevalence of the different grades of muscle echogenicity according to their evaluations (See Supplementary Table 1).

The US images and clips were obtained at the quadriceps muscle at the midpoint between the anterior superior iliac spine and the upper pole of the patella, as previously described (29). During clips acquisition, the probe was slowly moved 3 cm proximal and distal to the midpoint for a comprehensive exploration of the quadriceps muscle area. A MyLab C (Esaote Spa, Genoa, Italy) US system (frequency range 4–13 MHz, gain: 50 dB, depth 5 cm, or 6 cm in case of obese patients) and a MyLab9 XP (Esaote Spa, Genoa, Italy) broadband linear probe (frequency range 3–11 MHz, gain: 50 dB, depth 5 cm, or 6 cm in case of obese patients) were used for the acquisition of clips and images.

**FIGURE 1:**
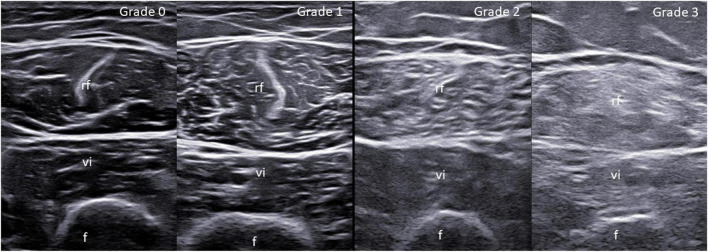
Visual semi-quantitative scale for the assessment of muscle echogenicity. Ultrasound (US) transverse scan images of the quadriceps muscle obtained at the midpoint between the anterior superior iliac spine and the upper pole of the patella. Grade 0 = normal (i.e., normal hypoechoic muscle); Grade 1 = mild (homogeneously distributed overall increase of the echogenicity involving ≤ one-third of the entire muscle tissue); Grade 2 = moderate (homogeneously distributed overall increase of the echogenicity involving > one-third but ≤ two-thirds of the entire muscle tissue); Grade 3 = severe (homogeneously distributed overall increase of the echogenicity involving > two-thirds of the entire muscle tissue). f, femur; rf, rectus femoris muscle; vi, vastus intermedius muscle.

**FIGURE 2:**
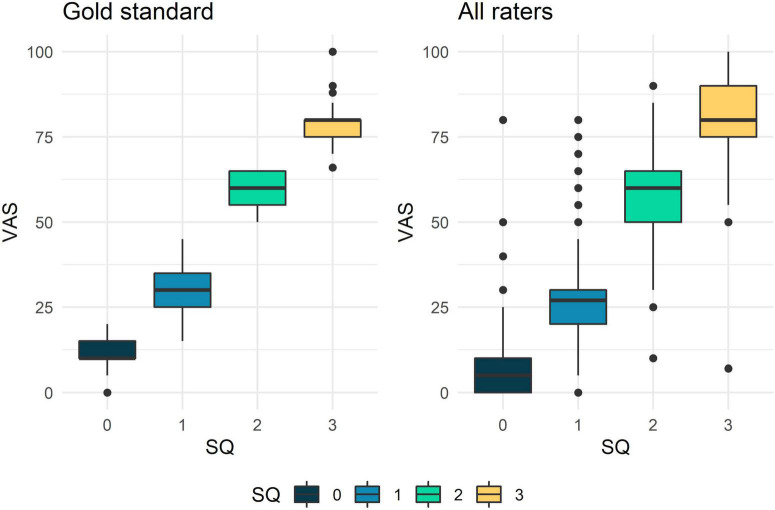
Boxplot of the joint semi-quantitative and quantitative (VAS echogenicity) ultrasound (US) scales distribution for muscle echogenicity. Box and whiskers plots showing the relationship between the semi-quantitative and the quantitative scores for muscle echogenicity. The upper and lower boundaries of the box represent the third and the first quartiles, respectively. The solid line in the box represents the median. Whiskers represent the maximum and the minimum values.

Participants, blinded to the patients diagnosis, were asked to score muscle echogenicity of quadriceps muscle (i.e., rectus femoris and vastus intermedius muscles) according to: (1) a visual semi-quantitative scale, recently developed by our research group (27) which grades muscle echogenicity from 0 to 3, where 0 = normal (normal hypoechoic muscle), 1 = mild (homogeneously distributed overall increase of the echogenicity involving ≤ one-third of the entire muscle tissue), 2 = moderate (homogeneously distributed overall increase of the echogenicity involving > one-third but ≤ two-thirds of the entire muscle tissue) and 3 = severe (homogeneously distributed overall increase of the echogenicity involving > two-thirds of the entire muscle tissue) (see Figure 1); (2) a visual quantitative scale (VAS echogenicity) ranging from 0 (black) to 100 (white). The same evaluation was repeated ≥6 weeks after the first one to assess the intra-observer reliability in scoring US images and clips. The online scoring spreadsheet which was used in the study is shown in Supplementary Figure 1. The distribution of the different grades of muscle echogenicity in patients with rheumatic diseases and healthy subjects is illustrated in Supplementary Table 2.

Muscle echogenicity of quadriceps muscle was also calculated in the 80 static images using ImageJ (version 1.53e) by a research assistant (SF), blind to the participants assessment of images and clips. ImageJ is a public-domain Java-based image-processing program that calculates the mean pixel grayscale intensity in a ROI using histogram analysis (30). ImageJ values of grayscale intensity range from 0 (black) and 255 (white). The rectus femoris and vastus intermedius muscles were included in the ROI to determine the mean pixel gray scale intensity. Particular attention was paid to include in the ROI only muscle tissues (i.e., without the surrounding fascia or cortical bone). Inter-observer reliability of ImageJ assessment resulted to be optimal in a recent article published by our research group (27).

### Statistical analysis

Prevalence of semi-quantitative rates was reported as counts and percentages. The inter and intra-rater reliability of the semi-quantitative scale was assessed by absolute agreement and Prevalence-Adjusted and Bias-Adjusted Kappa (PABAK), which was adopted to address prevalence imbalances in the rates. Intraclass Correlation Coefficient (ICC) was used to assess the inter and intra-reliability of VAS echogenicity. Kappa coefficients were interpreted according to Landis and Koch (31). The ICC was employed to inspect the association between the semi-quantitative scale and VAS echogenicity, whilst the Kendall’s Tau and Pearson’s Rho correlation coefficient were used for the correlation between semi-quantitative scale/VAS echogenicity and ImageJ.

## Results

Forty-four physicians (42 rheumatologists from 33 rheumatology centers and 2 radiologists from 2 radiology centers) from 13 countries participated in the study (see Table 1 for participants’ information).

**TABLE 1:** Main information of the participants in the study (*n* = 44).

Study participants		
Female gender, n (%)		20 (45.4%)
Years of experience in MSK US, median (IQR)		10.2 (7–15)
MSK US scans/month, median (IQR)		77.5 (30–110)
US scans of muscles/month, median (IQR)		4 (2–10)
Have you ever performed a muscle US scan?	Yes	38 (86.4%)
Why do you scan muscles?	Clinical reasons	28 (73.7%)
	Research purposes	2 (5.3%)
	Both	8 (21.1%)
**Country, n%**	**Rheumatology centers (*n* = 33)**	**Radiology centers (*n* = 2)**
Italy	8 (24.2%)	2 (100.0%)
Argentina	5 (15.2%)	/
Romania	4 (12.1%)	/
United Kingdom	3 (9.1%)	/
Greece	2 (6.1%)	/
Japan	2 (6.1%)	/
Portugal	2 (6.1%)	/
Colombia	2 (6.1%)	/
Brazil	1 (3.0%)	/
Canada	1 (3.0%)	/
Czechia	1 (3.0%)	/
Mexico	1 (3.0%)	/
Spain	1 (3.0%)	/

IQR, inter-quartile range; MSK, musculoskeletal; US, ultrasound.

The prevalence of the different grades of muscle echogenicity, as determined by the mean prevalence observed across raters, is reported in Table 2.

**TABLE 2:** Different grades of muscle echogenicity divided by images and clips as determined by the mean prevalence observed across raters.

All raters	Global (images + clips)	Images	Clips
Grade 0	18.3%	25.0%	18.2%
Grade 1	21.7%	23.8%	21.2%
Grade 2	27.5%	25.0%	25.2%
Grade 3	32.5%	26.2%	35.4%

Participants were asked to score a total of 80 images and 20 clips (global number of evaluations = 100).

### Inter-reliability assessment

As showed in Table 3, the overall (i.e., all grades together) global (i.e., images + clips) inter-reliability of the semi-quantitative scale was moderate [absolute agreement = 0.68 (0.68–0.69), PABAK = 0.58 (0.57–0.59)]. No considerable difference was observed between the assessment of images and clips.

**TABLE 3:** Reliability assessment of the visual semi-quantitative scale for muscle echogenicity.

	Images + clips (*n* = 100)		Images (*n* = 80)		Clips (*n* = 20)	
	*AA*	*PABAK*	*AA*	*PABAK*	*AA*	*PABAK*
**Inter-reliability assessment**						
Overall	0.68 [0.68–0.69]	0.58 [0.57–0.59]	0.69 [0.68–0.69]	0.58 [0.57–0.59]	0.67 [0.66–0.68]	0.55 [0.54–0.56]
Grade 0	0.66 [0.65–0.67]	0.43 [0.41–0.44]	0.67 [0.66–0.68]	0.42 [0.41–0.44]	0.61 [0.60–0.63]	0.20 [0.18–0.22]
Grade 1	0.60 [0.60–0.61]	0.24 [0.22–0.25]	0.60 [0.59–0.61]	0.21 [0.20–0.23]	0.61 [0.60–0.63]	0.29 [0.26–0.31]
Grade 2	0.64 [0.63–0.65]	0.23 [0.21–0.24]	0.64 [0.63–0.65]	0.22 [0.21–0.24]	0.65 [0.63–0.66]	0.39 [0.37–0.41]
Grade 3	0.82 [0.81–0.83]	0.37 [0.35–0.40]	0.83 [0.82–0.84]	0.39 [0.37–0.42]	0.78 [0.76–0.80]	0.10 [0.08–0.12]
**Intra-reliability assessment**						
Overall	0.78 [0.76–0.80]	0.71 [0.68–0.73]	0.78 [0.76–0.80]	0.70 [0.67–0.73]	0.76 [0.69–0.81]	0.67 [0.60–0.74]
Grade 0	0.78 [0.74–0.82]	0.69 [0.63–0.74]	0.77 [0.73–0.81]	0.67 [0.61–0.74]	0.75 [0.69–0.81]	0.61 [0.50–0.71]
Grade 1	0.59 [0.51–0.66]	0.47 [0.39–0.56]	0.56 [0.48–0.63]	0.42 [0.33–0.51]	0.74 [0.65–0.82]	0.65 [0.54–0.75]
Grade 2	0.55 [0.47–0.64]	0.44 [0.34–0.53]	0.55 [0.46–0.63]	0.42 [0.32–0.52]	0.68 [0.61–0.75]	0.52 [0.41–0.62]
Grade 3	0.68 [0.55–0.79]	0.63 [0.51–0.75]	0.71 [0.59–0.82]	0.66 [0.54–0.78]	0.70 [0.59–0.79]	0.61 [0.48–0.75]

AA, absolute agreement; PABAK, prevalence-adjusted, bias-adjusted kappa.

Values in square brackets are the 95% confidence intervals.

Lower reliability results (i.e., lower PABAK but absolute agreement consistent with the overall evaluation) were obtained when considering the single grades of the semi-quantitative scale separately. Grade 1 and grade 2 of the semi-quantitative scale showed the lowest absolute agreement and PABAK.

The reliability of VAS echogenicity was high, with no considerable differences between images and clips assessment [*images* + *clips* ICC = 0.80 (0.75–0.85); *images only* ICC = 0.80 [0.74–0.85]; *clips only* = ICC 0.84 [0.74–0.92]).

Since one of the possible reasons for the US changes of muscle quality is chronic inflammation, the inter reliability of US muscle echogenicity was also analyzed after excluding patients with osteoarthritis (*n* = 6) and fibromyalgia (*n* = 4). The inter-reliability of the semi-quantitative scale and VAS echogenicity without patients with osteoarthritis and fibromyalgia remained consistent with that of the whole population of rheumatic patients (see Supplementary Table 3).

Additional analyses were carried out including either the right or left side in patients in which a bilateral acquisition of the quadriceps muscle was obtained. The inter-reliability of the semi-quantitative scale and VAS echogenicity including such population resulted consistent with that of the total population (see Supplementary Tables 4, 5).

Finally, further analyses were performed by excluding those participants (i.e., raters) with no experience in the use of muscle US. This new analyses generated consistent results with those obtained in the whole group of raters (i.e., including those with no experience in the use of muscle US) (see Supplementary Table 6).

### Intra-reliability results

As illustrated in Table 3, the overall (i.e., all grades together) global (i.e., images + clips) intra-reliability of the semi-quantitative scale was substantial [absolute agreement = 0.78 (0.76–0.80), PABAK = 0.71 (0.68–0.73)]. No remarkable difference was noted between the assessment of images and clips.

Moderate to substantial intra-reliability was obtained when the single grades of the semi-quantitative scale were considered. Grade 1 and grade 2 of the semi-quantitative scale showed the lowest intra-reliability.

The reliability of VAS echogenicity remained high in the intra-observer assessment [*images* + *clips*: ICC = 0.88 (0.88–0.89); *images only* = ICC 0.88 (0.88–0.89); *clips only* = ICC 0.88 (0.88–0.89)].

The intra-reliability of the semi-quantitative scale and VAS echogenicity remained consistent when patients with osteoarthritis and fibromyalgia were excluded (see Supplementary Table 3), when only the right or left quadriceps muscle where considered in patients in which a bilateral US acquisition of the quadriceps muscle was obtained (see Supplementary Tables 4, 5), and when raters with no experience in the use of muscle US were excluded from the analyses (see Supplementary Table 6).

### Association between the semi-quantitative scale and continuous quantitative measurements for US muscle echogenicity, and their relationships with ImageJ

As shown in Figure 2, a substantial association was found between all participants’ evaluations using the semi-quantitative scale and VAS echogenicity [ICC = 0.52 (0.50–0.54)]. This corroborates the high correlation between the two visual scales that was obtained when the evaluations of the two rheumatologists who developed the images and clips dataset were taken into account (“gold standard,” *t* = 0.89, *p* < 0.01). For further details about numerical values see Supplementary Table 7.

In addition, a strong correlation was found between the participants evaluations using the semi-quantitative scale and VAS echogenicity and ImageJ analysis (*t* = 0.76 for the semiquantitative scale; *r* = 0.89 for VAS echogenicity). Similar good results were found when the “gold standard” assessment (i.e., the assessment of the two rheumatologists who developed the images and clips dataset) was considered (*t* = 0.76 for the semi-quantitative scale; *r* = 0.89 for VAS echogenicity).

## Discussion

The results of the current study demonstrated the overall good inter and intra-reliability of the US assessment of muscle echogenicity using an *ad hoc* developed dataset of US images and videos acquired in patients with rheumatic diseases.

This web-based exercise was carried out by a large group of rheumatologists and MSK radiologists who routinely perform US in their clinical practice, with a variable experience and training background in the US assessment of muscles (see Table 1).

The main objective of this study was to explore the reliability of two visual methods for the assessment of US muscle echogenicity, namely a semi-quantitative scale, which was recently developed by our research group (27), and a continuative quantitative measurement (VAS echogenicity), which was presented for the first time in this study. Both these two visual scales demonstrated an overall good inter and intra-reliability, with no remarkable difference between static images and clips assessment.

As shown in Table 3, variable grades of reliability were obtained when the single grades of muscle echogenicity of the semi-quantitative scale were evaluated. In the inter-reliability exercise, a grade 0 (i.e., normal muscle) and grade 3 (i.e., severe increase in muscle echogenicity) showed the highest, yet only moderate, degree of reliability. On the other hand, the intermediate grades of muscle echogenicity (i.e., grade 1 and grade 2 of the semi-quantitative scale) showed the lowest degree of reliability. Such low reliability results might be at least in part explained by the relatively small number of images and clips available for each single grade of the semi-quantitative scale. Indeed, the absolute agreement of the single grades of the semi-quantitative scale was comparable with the absolute agreement of the overall evaluation (i.e., all grades together). However, we acknowledge that the distinction between the different grades of the semi-quantitative scale may be difficult in those patients with “borderline” muscle echogenicity (e.g., between normal and mild, or mild and moderate), especially in the assessment of the intermediate grades of such scale (i.e., grade 1 and 2).

On the other hand, an overall higher degree of reliability for all the single grades of the semi-quantitative scale was obtained in the intra-observer assessment (i.e., substantial reliability for grade 0 and grade 3, moderate reliability for grade 1 and grade 2).

A significant correlation was observed between the semi-quantitative scale and VAS echogenicity. Both these visual scales grade muscle echogenicity abnormalities based on extent of muscle involved as opposed to degree of echo-intensity changes, as is the custom for grading US muscle echogenicity in myopathies/neuromuscular disorders (32). While a multifocal increase of muscle echogenicity could be observed in several neuromuscular disorders, such as muscular dystrophies, motor neuron disease (“moth-eaten appearance”) and inflammatory myositis, a homogeneous and broad involvement of muscle structures would be expected in patients with sarcopenia. For this reason, both the semi-quantitative scale and VAS echogenicity were developed by the current authors to score muscle echogenicity abnormalities as the extent of muscle area showing an increased echogenicity, rather than the degree of echo-intensity in a single “focal” area.

**FIGURE 3:**
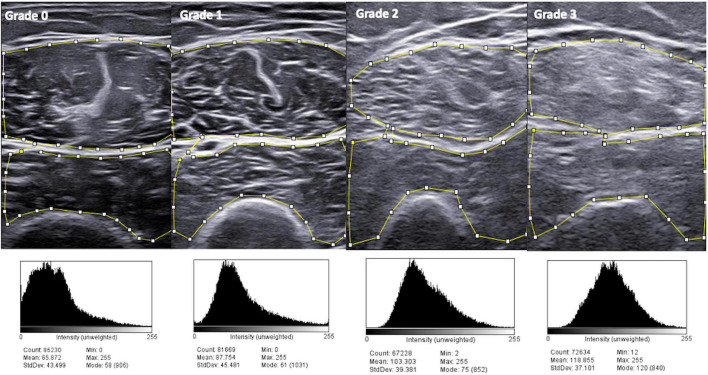
ImageJ analysis in patients with different grades of muscle echogenicity. Higher grades of the visual semi-quantitative scale for muscle echogenicity correspond to higher mean pixel analysis with ImageJ. During image analysis with ImageJ, particular attention was paid to include in the region of interest (ROI) only muscle tissues (i.e., without the surrounding fascia or cortical bone), which is the area included within the small squares and lines.

In addition, a significant association was found between both the semi-quantitative scale and continuous quantitative measurement and ImageJ (both with “all raters” and “gold standard” evaluations), which is a widely used software for processing and analyzing scientific images. Representative images of ImageJ analysis are reported in Figure 3. The main drawback of performing a software based evaluation is that this method is time consuming, other than being subjected to variations due to the fact that the areas of measurement (i.e., ROI) are defined by a human operator. In the current study, the software based evaluation with ImageJ required multiple steps: acquisition of US images on the US machine; upload of the US images from the US machine to a USB device and transfer to a computer/laptop; operator-based measurements of image echogenicity and acquisition of results. As described in the methods, the ImageJ operator of the current study included only muscle tissues in the ROI (i.e., without the surrounding fascia or cortical bone), which requires a careful and precise drawing of the borders defining the ROI (see Figure 3).

An increased muscle echogenicity is the result of the replacement of healthy muscle with fat (i.e., myosteatosis) rather than fibrosis and it is regarded as a reliable indicator of poor muscle quality (16). In addition, an increased US muscle echogenicity has been shown to be associated with muscle function and strength independently from a reduction of muscle mass, which is the main criteria for the diagnosis of sarcopenia (33–35). Therefore, US muscle echogenicity should be regarded as a reliable tool for the “early” detection of sarcopenia in patients with rheumatic diseases.

As acknowledged by the European Geriatric Medicine Society, US has a very promising role in the screening and “early” diagnosis of sarcopenia in patients susceptible to this condition (e.g., elderly populations, patients with chronic inflammatory diseases), given the ability of US to provide accurate assessment of muscle morphology and structure (i.e., muscle quality and muscle mass), good correlation with other imaging tools which are regarded as “gold standard” for the assessment of sarcopenia (e.g., MRI, CT scan, or DXA), patient’s bedside availability and relatively low costs (16, 19).

Imaging is one of the aspects that need to be considered in the diagnosis of sarcopenia. According to the European Working Group on Sarcopenia in Older People 2 (EWGSOP2), the diagnosis of sarcopenia should be made if loss of muscle mass (detectable with imaging) is accompanied by the reduction of patient’s muscle strength and/or impaired physical performance (1). In this context, US may play a key diagnostic/screening role in the “early” phase of sarcopenia, potentially identifying those patients who require further investigations (e.g., grip strength test, short physical performance battery) and, when a status of sarcopenia is confirmed, a specific management (i.e., referral to dedicated physiotherapy programs, potential use of drugs and/or supplements).

In a recent systematic literature review, the Outcome Measures in Rheumatology (OMERACT) group has highlighted the need for more evidence supporting the validity, reliability, and feasibility of quantitative methods for the evaluation of US domains of muscle involvement, including muscle echogenicity (36).

Using four pre-defined categories (i.e., normal, mild, moderate, and severe), the semi-quantitative scale which was recently developed by our group allows for a quick and intuitive classification of muscle echogenicity abnormalities (27). The current results suggest that a continuous quantitative measurement (VAS echogenicity) may represent a valid option to be used in alternative or in association with the semi-quantitative measurement, especially in those patients without a normal or clearly abnormal muscle echogenicity (i.e., grade 1 and grade 2 of the semi-quantitative scale). Even if more time-consuming than a visual assessment, the use of ImageJ analysis should be considered to obtain an objective, precise and patient-targeted measurement of muscle echogenicity, especially in those patients with intermediate grades of muscle echogenicity according to the proposed semi-quantitative scale. The good correlation emerged in the current study between a visual assessment (both using a semi-quantitative scale and a quantitative continuous measurement) and a software based evaluation suggests the opportunity to consider implementation of a digital measurement into the US machine.

The sensitivity to change of the two visual scales evaluated in the current study (i.e., responsiveness to interventions, such as use of drugs and/or supplements and/or adoption of regular physical exercise), represents an important aspect that needs to be further investigated. In addition, whether the reliability results of the two scales evaluated in the current study would be obtained in the assessment of muscles with different architecture and US appearance in comparison to the quadriceps muscle (e.g., gastrocnemius muscle or upper limb muscles), should be further explored (37).

The main limitation of the current study is that participants were asked to assess muscle echogenicity on static images and clips but did not perform the US examinations by themselves. This is an important aspect to consider also in light of the fact that the US images and clips might lose important quality information when they are converted to JPEG or AVI format compared to a “live” assessment on the US screen. Therefore, further patients-based studies are desirable. In addition, the US dataset of images and clips was generated by two operators using only two different US machines; this may potentially reduce the inter-observer variations if compared with a dataset generated by multiple operators using different US machines, thus limiting the generalizability of our results. Furthermore, the lack of comparison with a reference imaging tool for the assessment of muscle involvement, such as MRI or CT, should be considered as another limitation of the study. Indeed, this could have provided insights into the understanding of the US findings (e.g., differentiation between subclinical myositis, steroid myopathy, or sarcopenia), thus improving their validity. In this context, exploring the possible correlation between the US findings and a clinical score for sarcopenia (e.g., SARC-F) or the individuals measures of such condition (e.g., grip strength, short physical performance battery, or timed up and go test), could have clarified further the clinical relevance of US muscle echogenicity in the current population of rheumatic patients. Another limitation of the study is that the reliability assessments were carried out not taking into account the disease duration of the included patients, their age, and time of corticosteroid exposure. Indeed, all these aspects might determine changes in muscle echogenicity.

This study provides evidence in support of the reliability of US muscle echogenicity in patients with rheumatic diseases. The inter and intra-reliability of two recently developed scales for muscle echogenicity was evaluated, as well as their association with ImageJ, which is a widely used software for image analysis and processing. Therefore, this novelty is the main strength of the study.

In addition, data are presented from a multicenter study, which involved many experts in MSK US from several international countries.

## Conclusion

The results of this large, multicenter study support the reliability of US muscle echogenicity assessment in patients with rheumatic diseases, either using a visual semi-quantitative scale or a continuous quantitative measurement. US muscle echogenicity should be regarded as a reliable tool for the evaluation of changes of muscle quality in patients with rheumatic diseases, thus potentially representing a valuable tool for the “early” detection of sarcopenia in these patients.

## Data availability statement

The raw data supporting the conclusions of this article will be made available by the authors, without undue reservation.

## Ethics statement

The studies involving human participants were reviewed and approved by the local ethic committee (CERM n: 155/2021). The patients/participants provided their written informed consent to participate in this study.

## Author contributions

ADM, WG, and EF established the cohort. ADM designed the study and wrote the first draft of the manuscript. ADM and GS prepared the images and clips dataset. EM and ML developed the online exercise. GC, DR, CAS, and AZ carried out the statistical analysis. SF performed the ImageJ assessment. EC, CA, SA, AB, KB, MC, TC, DC, MAC, JA, GD, MD, ED, LD, ED, AE, DF, FF, AG, CH-D, RH, JH, DJ, JM, MVM, RM, MM, AM, JN, TO, JaR, MR, JoR, FS, CS, M-MT, ST, LV-R, CV-E, OV, PV, FV, GV, and JZH performed the reliability exercise. All authors contributed to revising the manuscript critically and approved the final version to be published.
