# Supplementary material for: Reliability assessment of ultrasound muscle echogenicity in patients with rheumatic diseases: Results of a multicenter international web-based study
Source: Front Med (Lausanne). 2023 Jan 17;9:1090468. doi: 10.3389/fmed.2022.1090468 (PMC9886677; doi:10.3389/fmed.2022.1090468)
Supplement: Supplementary file 1 [file Data_Sheet_1.PDF]

Supplementary Figure 1. Online scoring spreadsheet used in the study.

Image 17. Look at the image and answer the following questions.

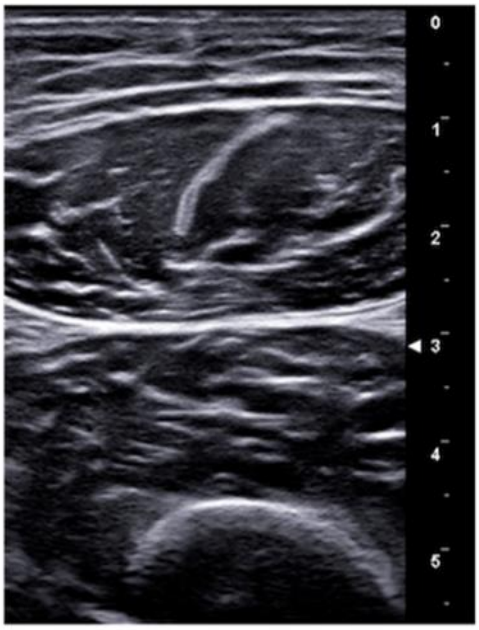

• According to the visual semiquantitative scale from 0 to 3, which grade of echogenicity would you assign to the image above?

○ 0

○ 1

○ 2

○ 3

• On a scale from 0 to 100 (0=black, 100=white), how would you rate the echogenicity of the image above?

Legend. Raters were asked to rate muscle echogenicity in the quadriceps muscle (i.e., rectus femoris and vastus intermedius).

Supplementary Table 1. Different grades of muscle echogenicity divided by images and clips as determined by the two rheumatologists who developed the images and videos dataset.

**Gold standard**	Global (images + clips)	Images	Clips
Grade 0	24.0%	25.0%	20.0%
Grade 1	24.0%	23.8%	25.0%
Grade 2	25.0%	25.0%	25.0%
Grade 3	27.0%	26.2%	30.0%

The final dataset was made by 80 static images and 20 clips (global n=100). 'Gold standard' refers to the two rheumatologists who developed the images and videos dataset.

Supplementary Table 2. Distribution of the grades of muscle echogenicity in patients with rheumatic diseases and healthy subjects.

	Grade 0	Grade 1	Grade 2	Grade 3	Total
**Systemic sclerosis**	3	5	5	11	24
**Axial Spondylarthritis**	4	6	7	4	21
**Rheumatoid arthritis**	2	5	4	3	14
**Systemic Lupus Erythematosus**	2	2	1	5	10
**Osteoarthritis**	1	1	4	2	8
**Fibromyalgia**	1	1	1	1	4
**Gout**	1	1	2	0	4
**CPPD**	1	0	1	1	3
**Healthy subjects**	9	3	0	0	12
**Total**	24	24	25	27	100

Legend. CPPD: calcium pyrophosphate deposition disease.

Supplementary Table 3. Reliability assessment of the visual semi-quantitative scale for muscle echogenicity excluding patients with osteoarthritis (n=6) and fibromyalgia (n=4).

INTER-RELIABILITY ASSESSMENT

**Semiquantitative scale**	Absolute agreement	PABAK
Global	0.68 [0.67-0.69]	0.58 [0.57-0.58]
Images	0.68 [0.68-0.69]	0.58 [0.57-0.59]
Clips	0.67 [0.66-0.68]	0.54 [0.53-0.56]

**VAS ECHOGENICITY**	ICC
Global	0.81 [0.76-0.85]
Images	0.80 [0.74-0.85]
Clips	0.85 [0.75-0.93]

INTRA-RELIABILITY ASSESSMENT

**Semiquantitative scale**	Absolute agreement	PABAK
Global	0.78 [0.76-0.80]	0.71 [0.68-0.74]
Images	0.76 [0.73-0.79]	0.69 [0.64-0.73]
Clips	0.75 [0.69-0.80]	0.67 [0.59-0.74]

**VAS ECHOGENICITY**	ICC
Global	0.88 (0.88-0.89)
Images	0.88 (0.88-0.89)
Clips	0.89 (0.89-0.90)

Legend. ICC: intraclass correlation coefficient; PABAK: prevalence-adjusted, bias-adjusted kappa. Values in square brackets are the 95% confidence intervals.

Supplementary Table 4. Reliability assessment of the visual semi-quantitative scale for muscle echogenicity including right quadriceps muscle only in subjects in which a bilateral acquisition of the quadriceps muscle was obtained (n=24 rheumatic patients and n=4 healthy subjects).

INTER-RELIABILITY ASSESSMENT

**Semiquantitative scale**	Absolute agreement	PABAK
Global	0.69 [0.68-0.70]	0.59 [0.58-0.60]
Images	0.69 [0.68-0.69]	0.58 [0.58-0.59]
Clips	0.70 [0.69-0.71]	0.51 [0.49-0.53]

**VAS ECHOGENICITY**	ICC
Global	0.81 [0.76-0.86]
Images	0.80 [0.74-0.86]
Clips	0.84 [0.74-0.93]

INTRA-RELIABILITY ASSESSMENT

**Semiquantitative scale**	Absolute agreement	PABAK
Global	0.77 [0.73-0.80]	0.69 [0.65-0.73]
Images	0.76 [0.73-0.79]	0.69 [0.64-0.72]
Clips	0.74 [0.67-0.80]	0.66 [0.57-0.73]

**VAS ECHOGENICITY**	ICC
Global	0.89 (0.89-0.89)
Images	0.89 (0.88-0.89)
Clips	0.89 (0.89-0.90)

Legend. ICC: intraclass correlation coefficient; PABAK: prevalence-adjusted, bias-adjusted kappa. Values in square brackets are the 95% confidence intervals.

Supplementary Table 5. Reliability assessment of the visual semi-quantitative scale for muscle echogenicity including left quadriceps muscle only in subjects in which a bilateral acquisition of the quadriceps muscle was obtained (n=24 rheumatic patients and n=4 healthy subjects).

INTER-RELIABILITY ASSESSMENT

**Semiquantitative scale**	Absolute agreement	PABAK
Global	0.69 [0.68-0.69]	0.58 [0.57-0.59]
Images	0.69 [0.69-0.70]	0.59 [0.58-0.60]
Clips	0.67 [0.65-0.68]	0.53 [0.51-0.54]

**VAS ECHOGENICITY**	ICC
Global	0.81 [0.76-0.86]
Images	0.81 [0.75-0.87]
Clips	0.83 [0.72-0.93]

INTRA-RELIABILITY ASSESSMENT

**Semiquantitative scale**	Absolute agreement	PABAK
Global	0.79 [0.77-0.81]	0.72 [0.69-0.74]
Images	0.78 [0.76-0.80]	0.71 [0.68-0.74]
Clips	0.74 [0.66-0.80]	0.66 [0.57-0.74]

**VAS ECHOGENICITY**	ICC
Global	0.89 (0.88-0.89)
Images	0.89 (0.89-0.89)
Clips	0.88 (0.88-0.89)

Legend. ICC: intraclass correlation coefficient; PABAK: prevalence-adjusted, bias-adjusted kappa. Values in square brackets are the 95% confidence intervals.

Supplementary Table 6. Reliability assessment of the visual semi-quantitative scale for muscle echogenicity excluding raters with no experience in the use of muscle ultrasound (n=6).

INTER-RELIABILITY ASSESSMENT

**Semiquantitative scale**	Absolute agreement	PABAK
Global	0.70 [0.69-0.70]	0.60 [0.59- 0.61]
Images	0.70 [0.69-0.71]	0.60 [0.59-0.61]
Clips	0.69 [0.68-0.70]	0.57 [0.57-0.59]

**VAS ECHOGENICITY**	ICC
Global	0.81 [0.76-0.85]
Images	0.81 [0.75-0.86]
Clips	0.84 [0.74-0.92]

INTRA-RELIABILITY ASSESSMENT

**Semiquantitative scale**	Absolute agreement	PABAK
Global	0.79 [0.76-0.80]	0.72 [0.69-0.74]
Images	0.78 [0.76-0.80]	0.71 [0.68-0.74]
Clips	0.77 [0.71-0.81]	0.68 [0.60- 0.76]

**VAS ECHOGENICITY**	ICC
Global	0.88 (0.88-0.89)
Images	0.88 (0.88-0.89)
Clips	0.89 (0.88-0.89)

Legend. ICC: intraclass correlation coefficient; PABAK: prevalence-adjusted, bias-adjusted kappa. Values in square brackets are the 95% confidence intervals.

Supplementary Table 7. VAS by Grade Summary statistics.

All raters

Grade	1st Quartile	Median	Mean	3rd Quartile
0	0	10	8.38	10
1	20	25	26.85	30
2	50	60	56.11	65
3	70	80	80.2	90

Gold standard

Grade	1st Quartile	Median	Mean	3rd Quartile
0	10	10	11.25	15
1	25	30	29.83	35
2	55	60	59.12	65
3	75	80	79.26	80

‘Gold standard’ indicates the evaluation made by the two rheumatologists who developed the final images and clips
